# Can intraoperative ultrasound visualize the closed reduction and elastic intramedullary nail fixation processes of double forearm fractures in children?

**DOI:** 10.1097/MD.0000000000024324

**Published:** 2021-03-26

**Authors:** Xiaodong Yang, Lang Li, Xueyang Tang, Bo Xiang, Tianfu Wen

**Affiliations:** a Department of Pediatric Surgery; b Department of Hepatic Surgery, West China Hospital, Sichuan University, Chengdu, China.

**Keywords:** children, closed reduction, double forearm fractures, elastic intramedullary nails, internal fixation, ultrasonography

## Abstract

To evaluate the feasibility of utilizing ultrasonography to monitor the fracture reduction and elastic intramedullary nail fixation processes in treating children with double forearm fractures. A retrospective analysis of 30 children with double forearm fractures treated at our hospital between January 2016 and July 2018. The children were aged 3 to 10 years. All patients were treated with closed reduction and internal fixation with elastic intramedullary nails using intraoperative ultrasound monitoring and intermittent radiographic imaging. The closed reduction and fixation were successful in all patients. The operation times ranged from 16 to 30 minutes, averaging 21 minutes. No neurovascular injuries occurred during closed reduction and nail insertion. Moreover, closed reduction was successful in the first attempt in 86.7% of patients. All patient outcomes were optimal, lacking serious complications during follow-ups. Intraoperative ultrasound monitoring can clearly show the shape and changes in fracture ends, distal growth plates, and surrounding soft tissues, and fracture reduction and passage of elastic nail processes at fracture ends during closed reduction; therefore, visualizing closed reductions can be achieved. The combination of intraoperative ultrasound and radiographic imaging can ensure operative effects and significantly reduce radiation exposure for both doctors and patients. An adequately powered prospective randomized trial is required to confirm our findings.

## 1 Introduction

Forearm fractures are a fracture type that commonly occurs in children, accounting for about 3% to 7% of all fractures in children. Nearly 90% of children can be treated by closed reduction and plaster fixation,^[1]^ but for some children, especially those who have not responded to conservative treatment, surgical treatment is required. Compared with distal metaphyseal fractures, forearm diaphyseal fractures have worse remodeling capacity and higher incidences of nonunion.

Certain unstable forearm fractures require surgical treatment.^[2]^ Elastic stable intramedullary nail fixation under intraoperative image intensifier monitoring represents a well established surgical stabilization method,^[3]^ which results in a certain level of radiation exposure to both doctors and patients. In this study, we experimented with intraoperative ultrasound monitoring during surgery, combined with C-shaped arm (C-arm) intermittent radiography to monitor reductions and internal fixations.

## 2 Patients and methods

Approval was obtained from the Ethics Committee of Sichuan University (No. 422/2015) and informed consent was obtained from the guardians of the patients for publication purposes. Among all the children treated with forearm fractures between January 2016 and July 2018, 30 patients were selected for this study according to the inclusion and exclusion criteria.

The inclusion criteria were:

1) Transverse fracture displacements exceeding 100%;
2) Fracture displacement angles in radius and ulna shaft fractures greater than 20 degrees;
3) Angulation greater than 10° or malrotation greater than 20° after closed reductions (via 3-dimensional computed tomography scan).

The exclusion criteria were:

(1) Monteggia, Galeazzi, pathological fractures, and children with polytrauma;
(2) Open fractures, fractures with nerve and vascular injuries, or with central nervous system injuries.

These 30 patients, 12 girls and 18 boys, aged from 3 to 10 years (mean age: 5.6 years), were treated by the same team of surgeons. All operations were conducted within 1 week post injury.

### 2.1 Surgical technique

**Figure 1:**
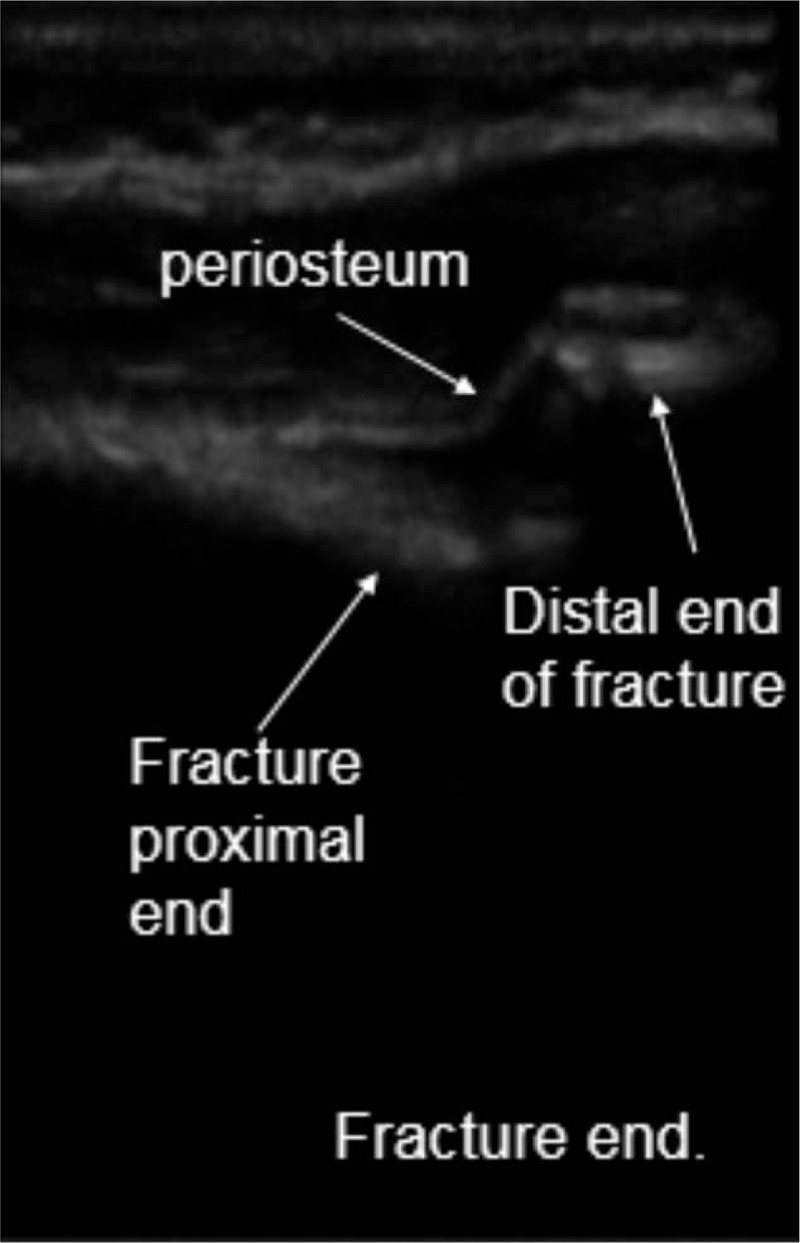
Intraoperative ultrasound to identify fracture sites.

**Figure 2:**
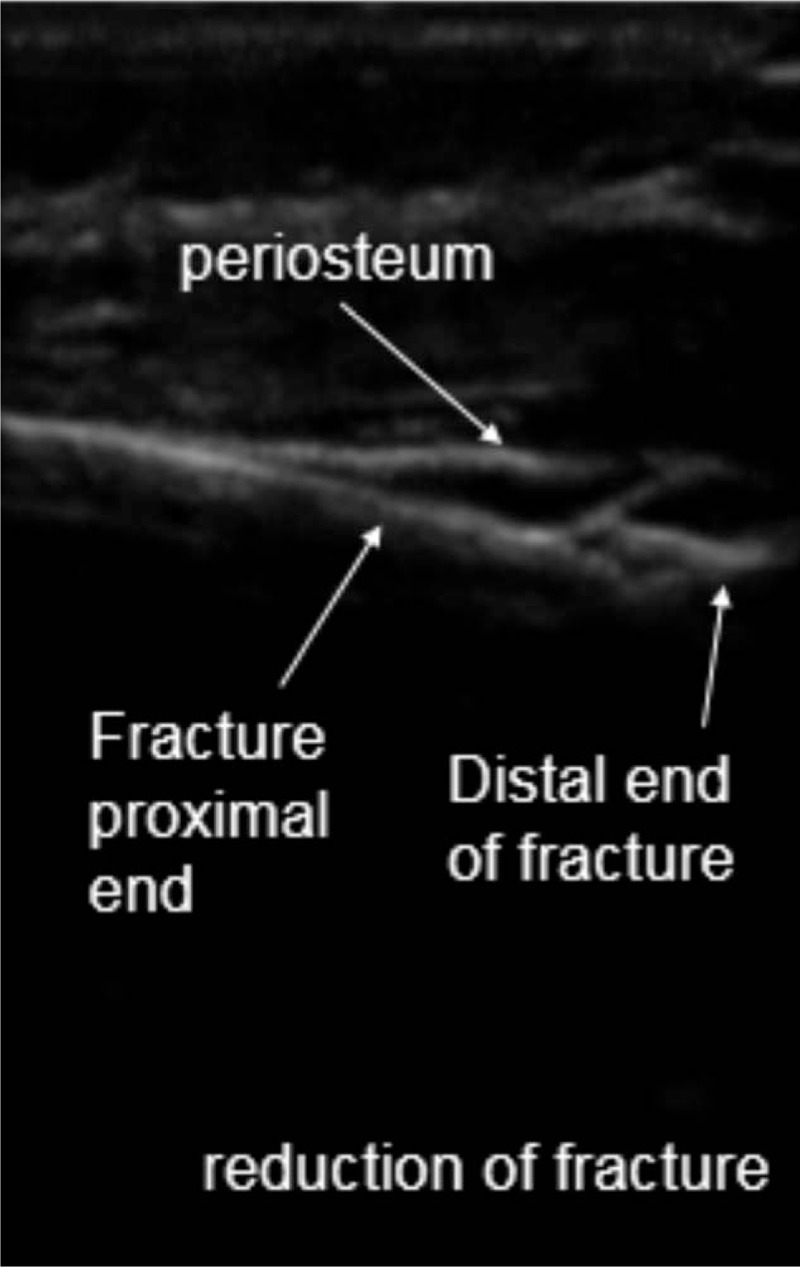
Conducting closed reduction while dynamically monitoring fracture fragments with ultrasound.

**Figure 3:**
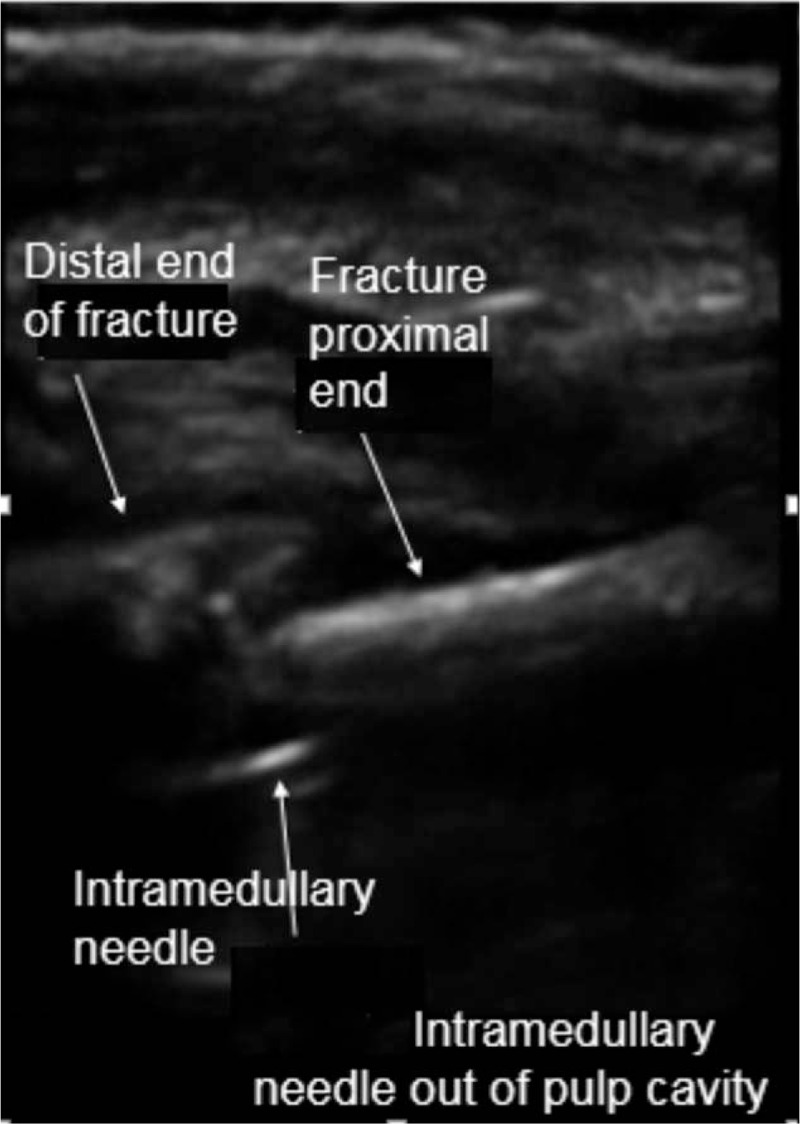
Placing nails correctly in the medullary cavity with guidance by ultrasound to detect whether the intramedullary nail exits the medullary cavity.

**Figure 4:**
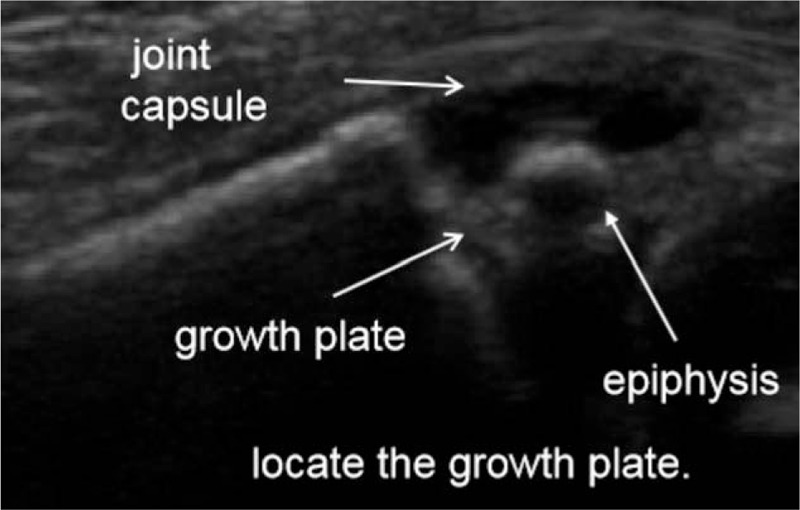
Ultrasound can be used to locate the growth plate.

**Figure 5:**
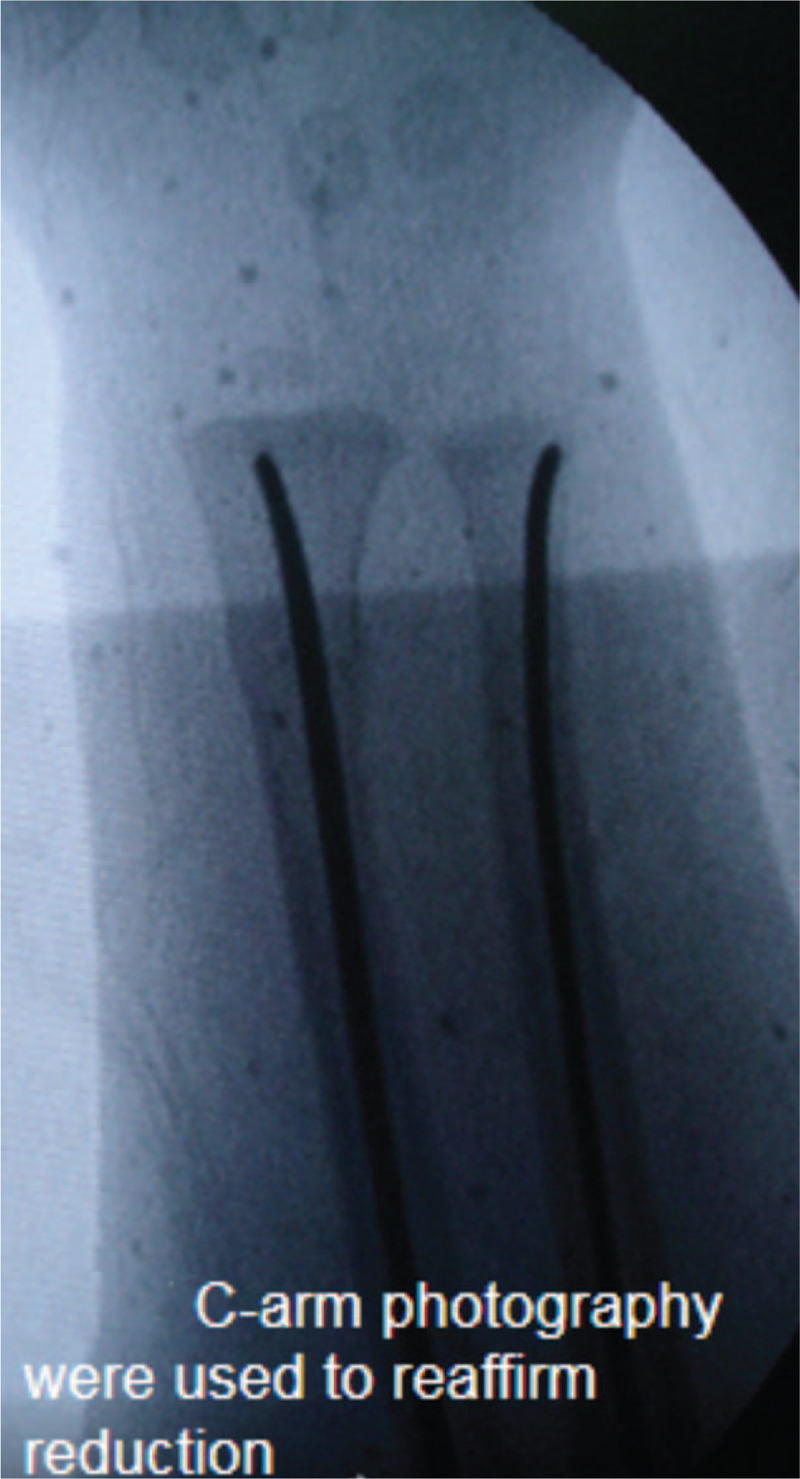
Images obtained by C-shaped arm imaging were used to reaffirm correct reduction and correct placement of the intramedullary nail.

Patients were placed in supine position. Operations were conducted using general anesthesia. A 6- to 13-MHz linear probe (HFL38x Linear Probe; M-Turbo, Sonosite, Bothell, WA) was used to scan the fractured bone from the distal fragment to the fracture site to identify the following anatomical landmarks: bone surface, and proximal and distal ends of the fracture (Fig. 1). Then, closed reduction was performed while the fracture was dynamically monitored via ultrasound (Fig. 2). Monitored via ultrasound, successful reductions were confirmed with the disappearance of the steps between the proximal and distal ends of the fracture, and the cortical bone exhibited a continuous straight line. Then, intramedullary nails were inserted into the proximal medullary cavity while the ultrasound probe scanned the fracture site to ensure that the intramedullary nails were placed inside the medullary cavity (Fig. 3 shows how to detect intramedullary nails exiting the medullary cavity). If no image detecting an intramedullary nail was found while conducting 360-degree ultrasound scanning of the fracture end, it indirectly proved that the intramedullary nails resided inside the marrow cavity. If the distal end of the fracture swung with the surgeon's hand at the same frequency while inserting the nail, it indicated that the intramedullary nail had entered the distal medullary cavity. We also may utilize image intensifiers to confirm that the intramedullary nail was inserted inside the medullary cavity after continuing to insert the nails about 3 cm, if necessary. After the intramedullary nails passed across the fracture site from the proximal medullary cavity into the distal medullary cavity, the ultrasound probe was utilized to locate the growth plate through ultrasound imaging and to mark the area of the growth plate on the skin using a marker, thus avoiding epiphyseal injuries in advance (Fig. 4). Finally, image intensifiers were employed to reaffirm reduction and internal fixation placement and help adjust reduction. Image intensifier imaging could be repeatedly utilized to detect internal fixation positions, if necessary (Fig. 5).

## 3 Results

With intraoperative ultrasound monitoring, closed reduction and fixation were successfully performed in all patients. The operation times ranged from 16 to 30 minutes, averaging 21 minutes. The image intensifier ultimately confirmed satisfactory reductions and internal fixations. No neurovascular injuries occurred during closed reduction and nail insertion. Moreover, closed reduction was successfully performed at first attempt in 26/30 (86.7%) patients.

**Figure 6:**
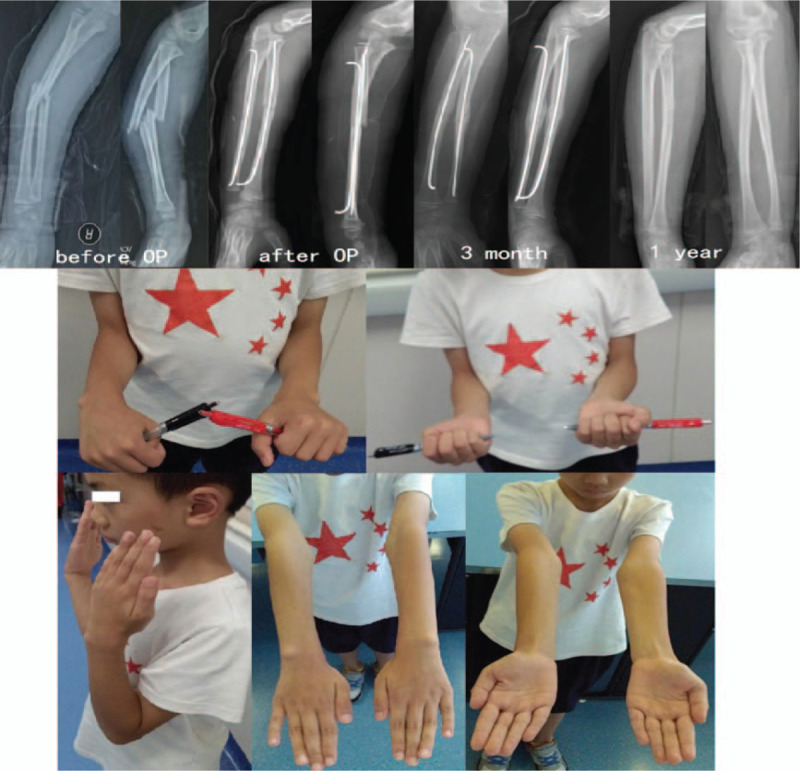
Typical case: roentgen ray imaging and function of forearm.

It took 9.8 weeks (range, 8–13 weeks) for the fracture to consolidate. No patients suffered from fracture site translations, malrotations of the radius, or nonunions. Implant removal was performed in all children at an average of 3.5 months (range, 3–6 months) post operation. According to Price grading system,^[4]^ the results were deemed excellent in 19 patients (63.3%), good in 9 patients (30%), and fair in 2 patients (6.7%). A typical case is shown in Fig. 6.

Postoperative complications occurred in 5 patients, including 3 patients with skin irritations due to nail end protrusion and 2 patients with superficial wound infections. All patients were treated with removing the nail and administering skin disinfection.

## 4 Discussion

According to Metaizeau's theory, the elastic intramedullary pin is the best option for patients with radius and ulna shaft fractures that need internal fixation.^[5]^ The traditional method is utilizing C-arm fluoroscopy to monitor shaft fracture reduction and internal fixation, which results in radiation exposure for surgeons and patients. Meanwhile, intermittent image intensifier images provide pictures of the fixed reduction position but lacks dynamic monitoring, which may lead to repetitive resets and multiple nail penetrations that can cause injury to endosteum and cortices.

To reduce radiation exposure and dynamically monitor fracture reduction and internal fixation processes, many researchers have reported the feasibility of ultrasound to diagnose fractures,^[6–8]^ especially in children. Ultrasound can also detect cartilage breaks.^[9]^ Mahaisavariya et al reported on adult intramedullary nail fixations under ultrasound monitoring.^[10]^ Yu et al have also reported similar findings,^[11]^ but no studies on children have been reported.

In this study, closed reductions and fixations were successfully applied in all patients. The average operation time was 21 minutes; moreover, closed reductions succeeded at the first attempt in 26/30 (86.7%) patients. No neurovascular injuries occurred during closed reductions and nail insertions.

The intraoperative ultrasound monitoring of children's forearm fractures and intramedullary nail fixations exhibits some major advantages:

1. It can improve the rate of fracture reduction at the first attempt. Intraoperative ultrasound could help locate fracture site and evaluate relative shifts between the broken ends of fractured bone. Repeated ineffective reductions could be avoided by utilizing dynamic ultrasound monitoring, and the reduction success rate significantly increased, so that additional fragmentation at the fracture site, induced by repeated reductions, can be avoided. Moreover, it can reduce radiation exposure in intraoperative image intensifier imaging, especially for the surgeon who perform numerous operations in a day.
2. It can reduce additional fragmentation of the fracture end: The ultrasound probe is placed at the area of the fracture site when the intramedullary nail passes from the proximal medullary cavity into the distal medullary cavity. A 3-dimensional image of the fracture site can be obtained with an ultrasound probe that scans the fractured bone in several planes. After the nail crossed the fracture site from the proximal into the distal medullary cavity, the ultrasound probe was used to locate the growth plate. Then, the area of the growth plate was marked on the skin using a marker. By determining the depth the nail was inserted, surgeons can avoid epiphyseal injuries in advance. Theoretically, dynamic monitoring with intraoperative ultrasound can help ensure that the intramedullary nail is accurately located in the medullary cavity to avoid causing additional fragmentation of the fracture end.
3. It can shorten operation time without increasing the total cost for patients. Dynamic monitoring of intraoperative ultrasound enables the reduction and internal fixation processes to be visualized. The entire operation is more manageable, so repeated unsatisfactory reductions can be avoided.

Song et al reported a case^[12]^ of a child with a ring finger flexion deformity after closed reduction and intramedullary nail fixation in whom they utilized conventional image intensifier monitoring. Postoperative ultrasound confirmed soft tissue interposition in the fracture gap, and then open reduction was performed. Thus, monitoring soft tissue is another advantage of ultrasound when compared to the roentgen ray images, avoiding soft tissue becoming entrapped in the fracture site.

### 4.1 Limitations

Some limitations inherent to this retrospective study could not be avoided. Additionally, the number of patients is too small, meaning the outcome may not necessarily be accurate. Thus, further randomized controlled studies are needed.

## 5 Conclusion

Ultrasound monitoring cannot completely replace intraoperative C-arm radiographic imaging. Only by combining the 2, significant improvements can be achieved for closed reduction monitoring. Ultrasound monitoring enables visualizing the entire operation in real-time, which adds additional control over the process. Especially in children, it can clearly show fracture fragments, the distal epiphyseal plate, and the soft tissue surrounding the fracture site during dynamic ultrasound investigations. It clearly shows the fracture reduction process and elastic intramedullary pin during closed reduction. A high rate (86.7%) for reductions in the first attempt, and short operation duration can be achieved. Therefore, we suggest that this method be administered to suitable patients.

## Author contributions

XDY wrote the manuscript. LL completed the conceptualization task. XYT investigated the patients. BX and TFW are the co-corresponding authors. They supervised the project.

**Conceptualization:** Lang Li, Tianfu Wen.

**Investigation:** Xueyang Tang.

**Project administration:** Bo Xiang.

**Supervision:** Tianfu Wen.

**Writing – original draft:** Xiaodong Yang.
